# Reactive Molecular Dynamics Simulation on Degradation of Tetracycline Antibiotics Treated by Cold Atmospheric Plasmas

**DOI:** 10.3390/molecules28093850

**Published:** 2023-05-01

**Authors:** Jinsen Guo, Yuantao Zhang

**Affiliations:** School of Electrical Engineering, Shandong University, Jinan 250061, China

**Keywords:** cold atmospheric plasmas, reactive molecular dynamics simulation, tetracycline antibiotics, bond breaking and formation, degradation

## Abstract

The abuse of tetracycline antibiotics (TCs) has caused serious environmental pollution and risks to public health. Degradation of TCs by cold atmospheric plasmas (CAPs) is a high efficiency, low energy consumption and environmentally friendly method. In this study, a reactive molecular dynamics (MD) simulation is applied to study the interactions of reactive oxygen species (ROS) produced in CAPs and TCs (including tetracycline (TC), oxytetracycline (OTC), chlortetracycline (CTC) and demeclocycline (DMC)). As revealed by the simulation data at the atomic level, the main reaction sites on TCs are the $C_{2}$ acylamino, the $C_{4}$ dimethylamine, the $C_{6}$ methyl group, the $C_{8}$ site on the benzene ring and the $C_{12a}$ tertiary alcohol. The interaction between ROS and TCs is usually initiated by H-abstraction, followed by the breaking and formation of the crucial chemical bonds, such as the breaking of C-C bonds, C-N bonds and C-O bonds and the formation of C=C bonds and C=O bonds. Due to the different structures of TCs, when the ROS impact OTC, CTC and DMC, some specific reactions are observed, including carbonylation at the $C_{5}$ site, dechlorination at the $C_{7}$ site and carbonylation at the $C_{6}$ site, respectively. Some degradation products obtained from the simulation data have been observed in the experimental measurements. In addition, the dose effects of CAP on TCs by adjusting the number of ROS in the simulation box are also investigated and are consistent with experimental observation. This study explains in detail the interaction mechanisms of degradation of TCs treated by CAPs with the final products after degradation, provides theoretical support for the experimental observation, then suggests optimization to further improve the efficiency of degradation of TCs by CAPs in applications.

## 1. Introduction

With the development of the modern synthetic chemical industry, a variety of synthetic organic pollutants (SOPs), including antibiotics, pesticides and detergents, have been detected in aquatic environments [1]. Antibiotics are secondary metabolites with anti-pathogen or other activity produced by microorganisms or other higher animals and plants [2,3]. Due to excellent broad-spectrum bactericidal properties and economy, they are widely used to treat human diseases and promote the growth of animals and plants [4,5,6]. However, the overuse of antibiotics poses threats to the environment [7,8]. Among antibiotics, TCs are the second-largest group and mainly include tetracycline (TC), oxytetracycline (OTC), chlortetracycline (CTC), demeclocycline (DMC) and other semi-synthetic tetracyclines [9,10]. TCs can specifically bind to the 30S subunit of the ribosome by blocking the binding of aminoacyl–tRNA at this position, thus inhibiting the growth of the peptide chain and affecting protein synthesis of the bacteria [11]. Effective against most gram-positive and gram-negative bacteria, TCs have rapidly become an indispensable antibacterial agent since their discovery [12]. Nevertheless, due to the essential properties of antibiotics, TCs can only be partially metabolized by humans and animals, and the vast majority of TCs are released into the environment as SOPs [13]. This greatly increases the risk of developing bacterial resistance and poses serious threats to human life [14,15]. Therefore, how to effectively degrade TCs has become the focus of attention.

So far, the commonly used degradation techniques for SOPs include physical degradation, chemical degradation and biodegradation methods. Physical degradation methods remove SOPs by transferring them from a liquid to a solid surface. These methods mainly include adsorption [16,17], filtration [18], coagulation and precipitation [19]. The advantages of physical degradation are environmental friendliness and fewer toxic byproducts. However, these methods cannot destroy the chemical structure of SOPs, and the cost of activated carbon for adsorption is high [20]. Chemical degradation methods mainly include ozone oxidation [21], photo-oxidation [22], Fenton oxidation [23,24] and electrochemical methods [25]. These methods involve the addition of specific chemical reagents into the effluent to produce chemical reactions or to degrade SOPs through oxidation reactions. They have the advantage of specific and effective degradation of SOPs through chemical reactions. Chemical degradation, however, is costly, and the addition of chemical reagents can produce more serious secondary pollution [26,27]. Biodegradation methods, including biofilm [28] and activated sludge methods [29], have received widespread attention for decades. These methods degrade SOPs to harmless small molecules by microbial metabolism. Their advantages are green environmental protection, no secondary pollution, large-scale application and low cost. Nevertheless, because of the antibacterial properties, TCs cannot be effectively removed by biodegradation [30].

Cold atmospheric plasmas (CAPs), as an advanced oxidation process, can effectively remove SOPs from water with high toxicity and low biological activity [31,32]. CAPs are a mixture of electrons, ions, atoms, molecules and active radicals. It can produce large amounts of reactive oxygen species (ROS) such as OH radicals, O atoms, $O_{3}$ molecules and $H_{2}O_{2}$ molecules, as well as reactive nitrogen species (RNS), such as nitric oxide (NO), nitrogen dioxide (${\mathrm{NO}}_{2}$), nitrous oxide ($N_{2}O$), and nitrogen trioxide (${\mathrm{NO}}_{3}$) [33,34]. These reactive species produced in CAPs can react with SOPs to degrade them, producing eco-friendly small molecules such as ${\mathrm{CO}}_{2}$ and $H_{2}O$. Due to the advantages of low energy consumption cost, high mineralization rate, low pollution and compatibility with existing treatment processes, degradation by CAPs has received remarkable attention [35].

In recent years, many experiments on the CAP treatment for SOPs in water have been carried out, and good results have been obtained. In a study by Aggelopoulos et al. [36], gas–liquid nanosecond-pulsed dielectric barrier discharge (nsp-DBD) plasma was used to treat effluent containing enrofloxacin (ENRO). Complete degradation of ENRO was finally achieved after 20 min at a pulse frequency of 200 Hz and a peak pulse voltage of 23.4 kV. Meanwhile, OH radicals were identified to contribute most to the degradation of ENRO through free radical scavengers, and a possible degradation pathway was proposed based on the intermediate products. Zhang et al. used gas-phase DBD plasma to degrade acetaminophen (APAP) in water [37]. Eventually they achieved 92% degradation of APAP after 18 min of treatment at 18 kV. Moreover, direct ozone attack and OH addition were proposed as the two main possible degradation pathways. Jose et al. investigated the degradation of chlorobenzene in aqueous solution by pulsed-power plasma [38]. After 12 min, 100% degradation of chlorobenzene occurred, and the dechlorination efficiency reached 85.3% after 20 min. It was also found that an increase in OH radical concentration leads to enhanced degradation of chlorobenzene. Therefore, OH radicals were determined as the main reactive species involved in the degradation of chlorobenzene.

Although a large number of experiments on the plasma degradation of SOPs have yielded good results, there is still a paucity of research on the fundamental mechanisms. Limited by the experimental conditions and equipment, researchers are speculating the reaction pathways between CAPs and SOPs by experimentally measured macroscopic products. It is difficult to observe their interaction processes at the atomic level and to distinguish the actual contribution of each oxygen species. This has caused difficulties in further studying how to apply CAP technologies to industry. The development of computer simulations has made it possible to solve this problem, where reactive molecular dynamics (MD) simulations can simulate the breaking and formation of chemical bonds during interactions, thus allowing visualization of the mechanisms of molecular interactions. Using reaction MD simulations, Tian et al. investigated the interaction processes between ROS generated in atmospheric plasma and fatty acids in vegetable oils, revealing the mechanisms of plasma interaction with vegetable oils and providing support for further in-depth studies of plasma medicine [39]. Zang et al. studied the degradation mechanisms of azithromycin molecules under the effect of non-thermal plasma using MD simulations and reactive force fields, which provided theoretical support for the subsequent experiments of degrading azithromycin [40].

Based on reactive MD simulations, the reaction processes of ROS produced in CAPs and TCs are investigated in this study. The interaction mechanisms of plasma degradation of TCs at the atomic level are deeply explored based on computational data, and the final products obtained from the computation agree well with the experimental observations, which partly supports the reliability and validation of the reactive MD simulation. In Section 2, the simulation results are discussed and analyzed. In Section 3, the computational method used in this study is explained in detail. The conclusion is summarized in Section 4.

## 2. Results and Discussion

The interaction processes of ROS and TCs have common and individual mechanisms. In this study, TC is selected as representative to display the common mechanisms, followed by the analysis of interactions between ROS and other TCs, respectively. Finally, dose effects are discussed.

### 2.1. Common Interactions between ROS and TCs

Upon the impact of OH radicals, a H atom in the $C_{2}$ primary amine is removed by an OH radical to form a $H_{2}O$ molecule. As a result of the interaction, the $C_{2}$ acylamino is destroyed, thereby reducing the activity of TC. The interaction mechanism between O atoms and the $C_{2}$ primary amine is similar to that of OH radicals, which also destroy the $C_{2}$ acylamino through H abstraction. In comparison, the effect of O atoms is more significant. An O atom can abstract two H atoms from the $C_{2}$ primary amine, so O atoms are about twice as effective as OH radicals. In the simulation, the highest probability of H-abstraction is observed at the $C_{2}$ acylamino, meaning that this reaction has the lowest energy barrier.

**Figure 1:**
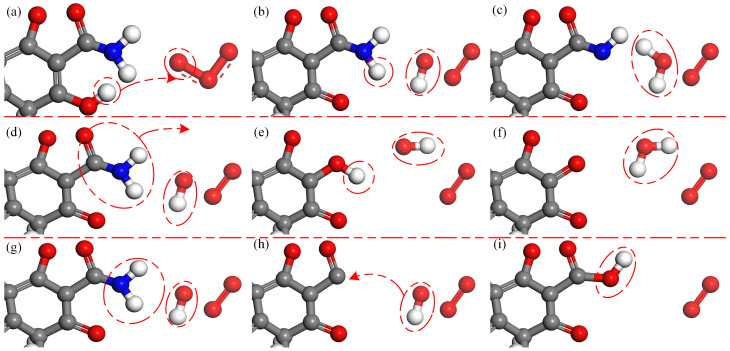
Breakage of N-H bond and formation of carbonyl and carboxyl upon the impact of $O_{3}$ molecules on TC. In the first type, a H atom is abstracted by an $O_{3}$ molecule from the hydroxyl seen in (**a**); then a H atom in the amino group is abstracted by the OH radical in (**b**,**c**). In the second type, the $C_{2}$ acylamino detaches from the $C_{2}$ site (**d**) and an OH radical attaches to this site (**e**), leading to the formation of carbonyl (**f**). In the third type, the $C_{2}$ primary amine detaches from acylamino (**g**) and an OH radical attaches to this site (**h**), finally leading to the formation of carboxyl (**i**).

**Figure 2:**
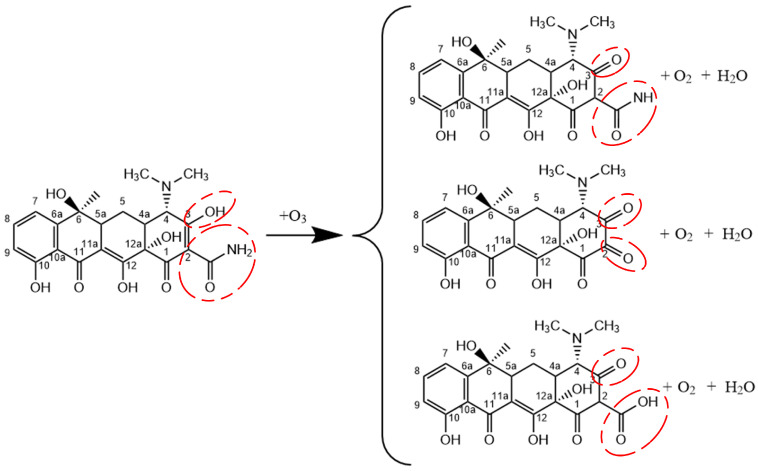
Reaction processes of $O_{3}$ molecules with the $C_{2}$ primary amine.

Figure 1 shows the interaction process between $O_{3}$ molecules and TC. The reaction positions are shown in red circles throughout this paper. As shown in Figure 1a–c, a H atom in TC is abstracted by an $O_{3}$ molecule to form an $O_{2}$ molecule and an OH radical. Subsequently, a H atom in the $C_{2}$ primary amine is abstracted by the OH radical to form a $H_{2}O$ molecule. The second reaction process is illustrated in Figure 1d–f. $O_{3}$ molecules have strong oxidizing properties, leading to the breaking of crucial bonds, such as C-C bonds and C-N bonds. Upon the impact of $O_{3}$ molecules, the C-C bond breaks, leading to detachment of the $C_{2}$ acylamino. Then, an OH radical attaches to the $C_{2}$ site and forms the carbonyl group after H-abstraction. The third reaction process is displayed in Figure 1g–i. Upon the impact of $O_{3}$ molecules, the C-N bond breaks, resulting in the detachment of the $C_{2}$ primary amine. In the following reaction, an OH radical attaches to this site to form the $C_{2}$ carboxyl group. The acylamino group after detachment forms ions such as ${\mathrm{NO}}_{3}^{-}$ upon oxidation by ROS, which can be detected in the experiments [41,42]. The molecular structures of reaction processes of $O_{3}$ molecules with the $C_{2}$ primary amine are shown in Figure 2. Three different products are shown in the figure, with H-abstraction being the most common reaction, especially in the case of 10 $O_{3}$ molecules, for which the value is close to 75%. Due to the high stability of amine, H-abstraction is the main reaction rather than the breaking of C-C bonds and C-N bonds. As the concentration of $O_{3}$ molecules increases, breaking of the C-C bond and the C-N bond increases close to 10% and 15%, respectively.

**Figure 3:**
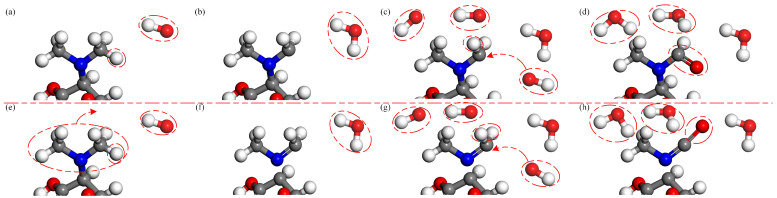
Formation of aldehyde group and detachment of dimethylamino group upon the impact of OH radicals on TC. In the first type, a H atom is abstracted by an OH radical from the methyl group in (**a**,**b**); then, another OH radical attaches to this site (**c**), leading to the formation of an aldehyde group (**d**). In the second type, the $C_{4}$ dimethylamine detaches from the $C_{4}$ site (**e**); then, a H atom is abstracted by an OH radical from the methyl group (**f**); finally, another OH radical attaches to this site (**g**), leading to the formation of carbonyl (**h**).

**Figure 4:**
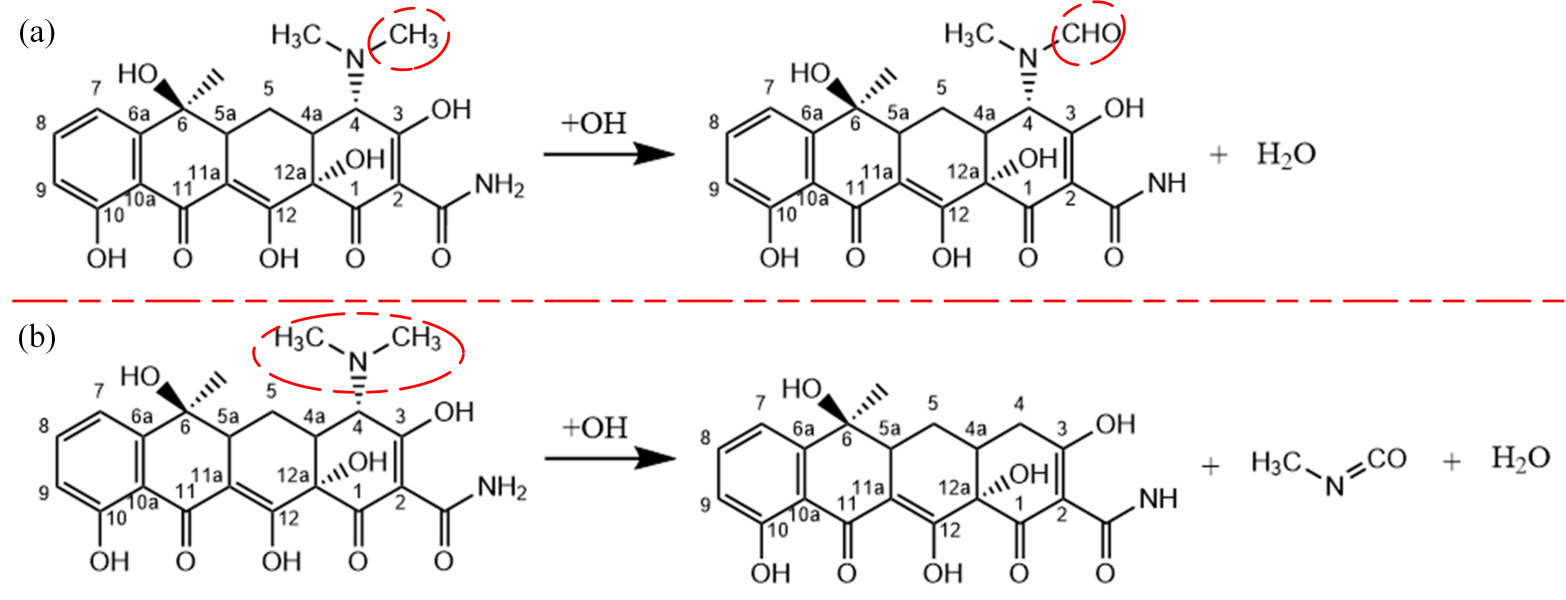
Reaction processes of OH radicals with the $C_{4}$ dimethylamine in (**a**,**b**).

Two reaction processes of OH radicals and the $C_{4}$ dimethylamine are illustrated in Figure 3. The first reaction process is displayed in Figure 3a–d. Among the dimethylamino group, the methyl group is the most vulnerable functional group to attack by reactive particles. When an OH radical approaches TC, it abstracts a H atom from the methyl group to form an $H_{2}O$ molecule. This increases instability of the $C_{4}$ dimethylamine. As the concentration of OH radicals increases, another OH radical abstracts a H atom from the methyl group. Subsequently, an OH radical attaches to this site to form an alcohol group. Finally, the H atom in the alcohol group is abstracted by an OH radical to form an aldehyde group. The second reaction process is shown in Figure 3e–h. Upon the impact of OH radicals, the C-N bond is broken and the dimethylamino group detaches from the $C_{4}$ site, followed by H-abstraction similar to the first reaction, leading to the formation of a carbonyl group. Then, the dimethylamino group is degraded to form small molecules, such as ${\mathrm{NH}}_{4}^{+}$, ${\mathrm{CO}}_{2}$ and $H_{2}O$. The interaction between the $C_{4}$ dimethylamine with O atoms and $O_{3}$ molecules is similar to that of OH radicals. The corresponding results have also been observed in experiments [43]. As the experiment progresses, the conductivity of the solution in the experiment increases due to the production of inorganic ions such as ${\mathrm{NH}}_{4}^{+}$. Figure 4 exhibits the molecular structures of reaction processes of OH radicals with the $C_{4}$ dimethylamine.

**Figure 5:**
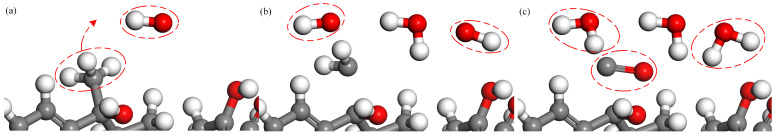
Formation of carbon monoxide upon the impact of OH radicals on TC. The $C_{6}$ methyl group detaches from the $C_{6}$ site (**a**); then, the produced methyl group react with OH radicals (**b**), yielding the formation of carbon monoxide (**c**).

**Figure 6:**
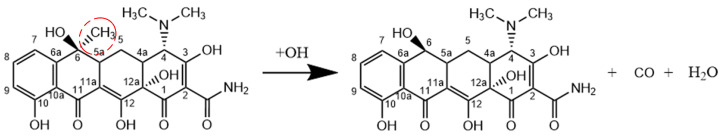
Reaction process of OH radicals with the $C_{6}$ methyl group.

The reaction process of OH radicals with the $C_{6}$ methyl group is shown in Figure 5. Upon the impact of OH radicals, the C-C bond is broken at the $C_{6}$ site, leading to detachment of the $C_{6}$ methyl group. This effectively destroys the activity of the TC molecule. After the removal of the $C_{6}$ methyl group, a series of H-abstraction and hydroxyl addition reactions take place on it upon the impact of OH radicals, and the $C_{6}$ methyl group is eventually oxidized into small molecules such as CO and ${\mathrm{CO}}_{2}$. O atoms and $O_{3}$ molecules have similar effects at this site. The molecular structure of the reaction process of OH radicals with the $C_{6}$ methyl group is displayed in Figure 6.

As one of the most common active functional groups, benzene rings have always been a focus of researchers. Upon the impact of OH radicals, the H atom at the $C_{8}$ site on the benzene ring is abstracted, and then an OH radical attaches to this site to form an alcohol group. Another OH radical abstracts the H atom in the alcohol group to form a $H_{2}O$ molecule, resulting in the formation of the carbonyl group at the $C_{8}$ site. O atoms and $O_{3}$ molecules can do the same. This interaction process destroys the structure of the benzene ring, which affects the activity of the TC molecule. In the simulation, we observed that oxidation of the benzene ring occurred after the reactions mentioned earlier, indicating that the activity of the benzene ring is less than that of other functional groups.

**Figure 7:**
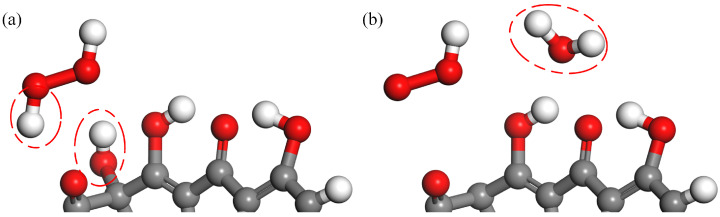
Detachment of hydroxyl upon the impact of $H_{2}O_{2}$ molecules on TC. The $C_{12a}$ tertiary alcohol reacts with an $H_{2}O_{2}$ molecule (**a**), leading to the detachment of hydroxyl (**b**).

**Figure 8:**
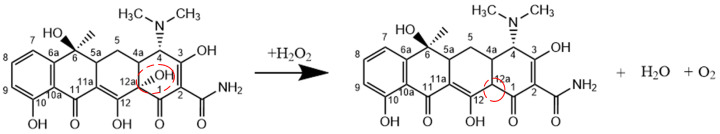
Reaction process of $H_{2}O_{2}$ molecules with the $C_{12a}$ tertiary alcohol.

In Figure 7, the interaction process between $H_{2}O_{2}$ molecules with the $C_{12a}$ tertiary alcohol is shown. Unlike the interaction mechanisms of other ROS, $H_{2}O_{2}$ molecules react with each other to form $HO_{2}$ radicals and H atoms before reacting with TC molecules. Upon the impact of the formed H atoms, the $C_{12a}$ tertiary alcohol is abstracted to form a $H_{2}O$ molecule, which destroys the $C_{12a}$ tertiary alcohol functional group, leading to the degradation of TC molecules effectively. Figure 8 exhibits the molecular structure of the reaction process of $H_{2}O_{2}$ molecules with the $C_{12a}$ tertiary alcohol. The reaction process of $O_{3}$ molecules with the $C_{12a}$ tertiary alcohol is different from that of $H_{2}O_{2}$ molecules. An $O_{3}$ molecule first abstracts a H atom from the $C_{12}$ tertiary alcohol, leading to the breaking of the $C_{12}$-$C_{12a}$ bond. Meanwhile, another $O_{3}$ molecule abstracts a H atom from the $C_{12a}$ tertiary alcohol, which eventually leads to the formation of C=O bonds at the $C_{12}$ and the $C_{12a}$ sites; also, the TC molecule unrings. The TC molecule structure and the $C_{12a}$ tertiary alcohol are destroyed effectively from the simulation.

### 2.2. Respective Individual Interactions between ROS and Other TCs

The difference between the structure of OTC and that of TC is the $C_{5}$ secondary alcohol on OTC. The H atom in the $C_{5}$ secondary alcohol has a low energy barrier and is more vulnerable to attack by OH radicals. Upon the impact of OH radicals, the H atom in the $C_{5}$ secondary alcohol is abstracted by an OH radical to form an $H_{2}O$ molecule, leading to the formation of a carbonyl group. This reaction effectively destroys the activity of the $C_{5}$ secondary alcohol on OTC, which is helpful for the degradation of OTC.

**Figure 9:**
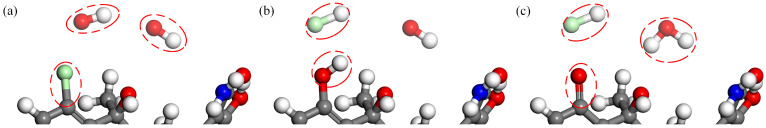
Formation of hydrogen chloride molecule upon the impact of OH radicals on CTC. The $C_{7}$ chloride group detaches from the $C_{7}$ site (**a**); then, an OH radical attaches to this site (**b**), finally leading to the formation of hydrogen chloride molecule and carbonyl (**c**).

**Figure 10:**
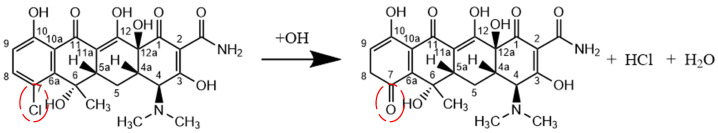
Reaction process of OH radicals with the $C_{7}$ chloride group on CTC.

Compared with TC, the unique reactive functional group of CTC is the $C_{7}$ chloride group, as shown in Figure 9. The chloride group is a typical toxic functional group in molecules, so its removal can effectively degrade CTC. Indeed, upon the impact of OH radicals, the chlorine group at the $C_{7}$ site detaches and reacts with a H atom to form an HCl molecule. Hydroxyl addition and carbonylation occur at the $C_{7}$ site subsequently. This effectively destroys the activity of the $C_{7}$ chloride group and plays a crucial role in the degradation of CTC. Figure 10 shows the molecular structure of the reaction process of OH radicals with the $C_{7}$ chloride group on CTC.

**Figure 11:**
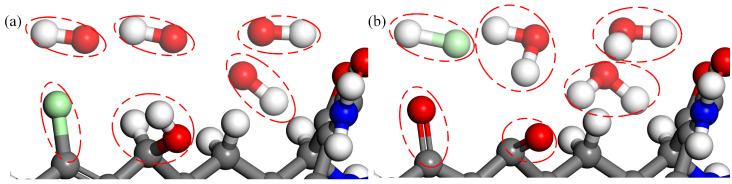
Formation of hydrogen chloride molecule and carbonyl upon the impact of OH radicals on DMC. The $C_{7}$ chloride group detaches from the $C_{7}$ site, accompanied by H abstraction in the $C_{6}$ secondary alcohol (**a**), leading to the formation of hydrogen chloride molecule and carbonyl (**b**).

**Figure 12:**
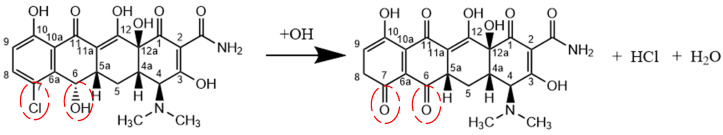
Reaction process of OH radicals with the $C_{6}$ secondary alcohol and the $C_{7}$ chloride group on DMC.

The structure of DMC is similar to that of CTC, with the exception that DMC lacks the $C_{6}$ methyl group, as displayed in Figure 11. Therefore, OH radicals can also result in the detachment of the $C_{7}$ chloride group, followed by hydroxyl addition reaction and H-abstraction at this site. Meanwhile, the H atom in the $C_{6}$ secondary alcohol is abstracted by OH radicals to form a carbonyl group. The molecular structure of the reaction process is shown in Figure 12.

Through calculation and analysis, it is found that ROS produced in CAPs can have a variety of common and individual interactions with TCs. In the simulation, ROS first abstract H atoms from TCs; then, a series of reactions such as hydroxylation, dehydroxylation, carbonylation, carboxylation and aldehyde occur subsequently. ROS produced in CAPs can effectively destroy crucial active functional groups in TCs, indicating that CAPs can effectively degrade TCs. It is worth mentioning that the reaction processes between ROS and active functional groups of TCs occur synchronously rather than sequentially; thus, the reaction products observed in the experiment contain all the reaction results that have occurred. In experiment [42], detachment of the $C_{4}$ dimethylamine and the $C_{6}$ methyl group on TC is observed. In experiment [41], formation of the $C_{2}$ carboxyl group is observed. In experiment [43], carbonylation of the $C_{5}$ secondary alcohol on OTC is observed. Compared with the degradation experiments of four TCs, good corresponding results are obtained between simulation results and reaction products, which verifies the rationality and accuracy of the calculation.

### 2.3. Dose Effects of Interactions between ROS and TCs

**Figure 13:**
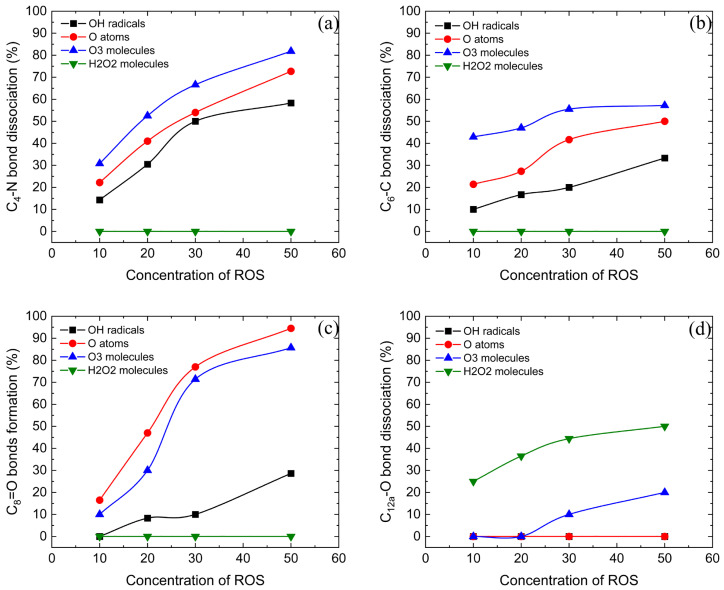
Variation in fractions of $C_{4}$–N bond dissociation (**a**), $C_{6}$–C bond dissociation (**b**), $C_{8}$=O bond formation (**c**) and $C_{12a}$–O bond dissociation (**d**) upon the impact of four ROS on TCs.

In the experiments of plasma degradation of TCs, the concentration of ROS can be changed by varying various chemical doses and discharge parameters, such as the concentration of catalyst, voltage amplitude, discharge frequency, etc. [41,42,43]. In experiment [41], by increasing the concentration of ${\mathrm{TiO}}_{2}$ catalyst, the formation of the electric field channel and the penetration of light radiation could be effectively promoted. This resulted in the increased production of ROS and an improved degradation rate of TC. When the ${\mathrm{TiO}}_{2}$ concentration reached 1.5 ${\mathrm{gL}}^{-1}$, the degradation rate of TC was up to 80.1%. In experiment [43], by adjusting the discharge voltage amplitude, the concentration of ROS in the plasma vessel was changed. This led to different degradation rates of up to 98.1% for OTC, with the optimal voltage amplitude measured as 18kV. In the simulation, different numbers of ROS are added to the constructed reaction cell to simulate different concentrations of ROS in the experiments. Through extensive simulated calculations of interactions between ROS and TCs, the dose effects are summarized, as shown in Figure 13.

Figure 13a exhibits the variation between the breaking of the $C_{4}$–N bond and the concentration of ROS. All three ROS (OH radicals, O atoms and $O_{3}$ molecules) can effectively break the $C_{4}$–N bond, and the rate of $C_{4}$–N bond breaking increases rapidly with the increase in ROS concentration. Among them, $O_{3}$ molecules have the strongest effect on the breaking of $C_{4}$–N bonds, up to 81.8%, followed by O atoms and OH radicals. On the contrary, $H_{2}O_{2}$ molecules have no effect on the breaking. The rate of $C_{6}$–C bond breaking is illustrated in Figure 13b. Three types of ROS (OH radicals, O atoms and $O_{3}$ molecules) can result in the breaking of the $C_{6}$–C bond, and the rate of $C_{6}$–C bond breaking gradually increases with the increase in ROS concentration. Among them, $O_{3}$ molecules have the strongest effect on the $C_{6}$–C bond breaking, up to 57.2%. Meanwhile, $H_{2}O_{2}$ molecules have no effect on the breaking of the $C_{6}$–C bond. Figure 13c displays the formation of the $C_{8}$=O bond upon the impact of different types and concentration of ROS. Three varieties of ROS (OH radicals, O atoms and $O_{3}$ molecules) can promote the formation of the $C_{8}$=O bond, and the rate of $C_{8}$=O bond formation also increases with the increase in ROS concentration. Among them, O atoms and $O_{3}$ molecules have a strong effect on the formation of the $C_{8}$=O bond—up to 94.5% and 85.7%, respectively—while OH radicals have a general effect, up to 28.6%. $H_{2}O_{2}$ molecules still have no effect on the formation of the $C_{8}$=O bond. Figure 13d shows the breaking of the $C_{12a}$–O bond upon the impact of ROS. Different from the previously mentioned reactions, $H_{2}O_{2}$ molecules have the best effect on the breaking of the $C_{12a}$–O bond, with a rate up to 50%. $O_{3}$ molecules also play a certain role. It can be noted that the $C_{12a}$–O bond is broken by forming the $C_{12a}$=O bond, with the rate of breaking up to 20%. The other two types of ROS (O atoms and OH radicals) do not contribute to $C_{12a}$–O bond breaking.

As can be seen from these simulation data, $O_{3}$ molecules have the most significant effect on the breaking of the $C_{4}$–N bond and the $C_{6}$–C bond in TCs. This indicates that $O_{3}$ molecules, compared with O atoms, OH radicals and $H_{2}O_{2}$ molecules, are the ROS that can most promote the breaking of the C-C bonds and the C-N bonds. This is consistent with the effect of $O_{3}$ molecules speculated in the previous experiments. O atoms perform better in the formation of the $C_{8}$=O bond, indicating that O atoms play the most crucial role in the oxidation of the benzene ring. Unfortunately, the oxidation of O atoms cannot be well measured due to their great reactivity not only with TCs but also with water, which highlights the significance of MD simulation. OH radicals play an important role in the breaking and formation of crucial chemical bonds, so they are indispensable ROS in the plasma degradation of TCs, which has been well observed in the experiments. The interactions between $H_{2}O_{2}$ molecules and TCs are different from those of the other three ROS. They mainly play an important role in the detachment of the $C_{12a}$ tertiary alcohol, which has often been ignored in previous experiments and can be well observed in MD simulation. The common effect of these ROS is that the rate of breaking and forming crucial chemical bonds in TCs increases with the increase in ROS concentration. In other words, the degradation rate of TCs increases with the increase in ROS concentration, which is consistent with the trend of reactions observed in the experiments.

Subsequently, the simulation results are compared with the degradation pathways postulated in the experiments. As shown by the simulation results in Figure 13, the impact of ROS can lead to the breakage of the $C_{4}$–N bond, the breakage of the $C_{6}$–C bond, the formation of the $C_{8}$=O double bond, and the breakage of the $C_{12a}$–O bond, respectively, which are also reflected in the experimentally determined intermediates. The breakage of the $C_{4}$–N bond leads to the detachment of dimethylamine, which causes the formation of ${\mathrm{NH}}_{4}^{+}$ and ${\mathrm{NO}}_{3}^{-}$ with the subsequent action of ROS. This is consistent with the experimental intermediates (O12 and O16) [41,43]. The breakage of the $C_{6}$–C bond leads to the detachment of methyl group, after which the shed methyl group is oxidized to small molecules such as ${\mathrm{CO}}_{2}$, which is detected in the experiments (a product with *m*/*z* = 274) [42,44]. An OH addition reaction at the $C_{8}$ site is also observed in the experiments (a product with *m*/*z* = 477b), similar to the formation of the $C_{8}$=O double bond [45]. Breakage of the $C_{12a}$–O bond leads to detachment of the hydroxyl group, and formation of the product is consistent with the experiments (O16) [43]. In addition, upon the impact of ROS, the $C_{2}$ acylamino group also detaches and a carboxyl or carbonyl group is formed at the $C_{2}$ site. Intermediate products containing the $C_{2}$ carboxyl group are also observed in the experiments (a product with *m*/*z* = 364) [41,44]. The comparison of simulation results and experimental products shows the realism and reliability of the simulations.

## 3. Molecular Dynamics Simulations

**Figure 14:**
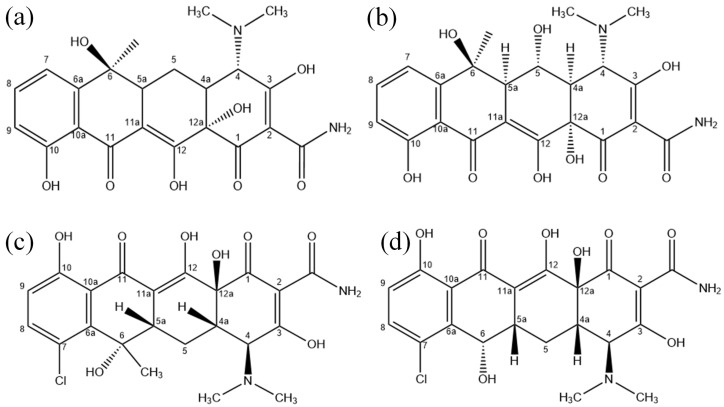
Molecular structures of tetracycline (**a**), oxytetracycline (**b**), chlortetracycline (**c**) and demeclocycline (**d**).

In recent years, CAPs have attracted more and more attention due to their excellent degradation effects. According to different discharge methods, they can be mainly divided into DBD plasma and atmospheric-pressure plasma jets (APPJs) [46]. In plasma degradation experiments of TCs, the gas is fully or partially ionized by injecting energy through a high-voltage AC power supply and a plasma generator, generating large amounts of primary charged particles of ROS and RNS. These charged particles react in the gas phase to produce long-lived neutral radicals that diffuse into the gas–liquid interface and the liquid [47]. The interaction of radicals with liquids produces more species of ROS and RNS, with the most important particles of ROS being OH radicals, O atoms, $O_{3}$ molecules and $H_{2}O_{2}$ molecules. In experiments, it has been concluded that OH radicals, $O_{3}$ molecules and $H_{2}O_{2}$ molecules are very effective at degrading TCs, especially OH radicals and $O_{3}$ molecules, by adding specific reactive particle scavengers for determination [41,42,43]. Since O atoms are very reactive with both TCs and water, they cannot be measured well experimentally. However, it has been found in previous simulations that O atoms have a good effect on the degradation of organic pollutants, so reactions between O atoms and TCs have also been simulated [40,48]. In fact, there are other factors affecting the degradation effect in the experiments, such as the influence of RNS, electric fields and UV, but it has been observed that ROS, especially OH radicals, play a crucial role in the degradation of TCs [49]. As shown in Figure 14, there are many active functional groups in the molecular structures of TCs, including the $C_{2}$ acylamino, the $C_{4}$ dimethylamine, the $C_{6}$ methyl group, the benzene ring and the $C_{12a}$ tertiary alcohol. OTC, CTC and DMC have their own active functional groups, namely the $C_{5}$ secondary alcohol, the $C_{7}$ chloride group and the $C_{6}$ secondary alcohol, respectively. These functional groups are attacked mainly because these are all electron-rich functional groups. Therefore, they are more reactive and will be directly attacked by ROS [50]. Indeed, the destruction of active functional groups by ROS is the most efficient way to degrade TCs [51]. Since the exact mechanisms of interaction are still unclear, the interaction processes between ROS and TCs are investigated in this study.

In order to investigate the interaction processes between TCs and ROS more accurately and to summarize the interaction mechanisms, four different natural tetracyclines (TC, OTC, CTC and DMC) are selected in the present computational study. Before starting the simulation, the simulation model is established according to the structure in Figure 14. The COMPASS II force field is used to optimize the geometry and dynamics of the four TCs. The purpose is to achieve a stable state with minimum energy and to prevent the non-reactive structural deformation of TCs during the simulation.

**Figure 15:**
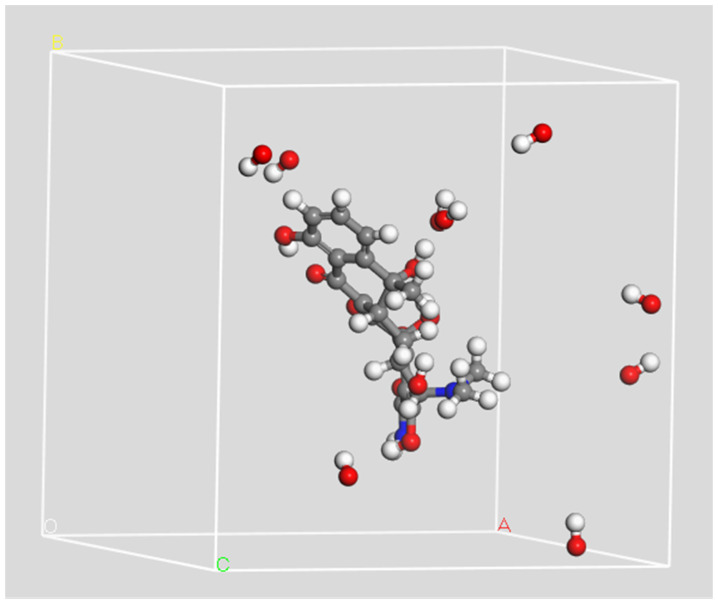
Example of simulation box consisting of a tetracycline and 10 OH radicals with periodic boundary conditions applied in three directions.

All the simulation processes in this study are carried out in the NVT ensemble (constant number of particles (N) under constant volume (V) and constant temperature (T)); T = 300 K. The optimized structural model of TCs and different numbers (10, 20, 30 or 50) of ROS (OH radicals, O atoms, $O_{3}$ molecules and $H_{2}O_{2}$ molecules) are placed in a simulation box of 20 Å × 20 Å × 20 Å, as shown in Figure 15. Atoms of C, H, O, N and Cl are represented by gray, white, red, blue and green spheres, respectively. This ratio of ROS to TCs in the simulation is much higher than the ratio of ROS to TCs in the actual reaction. Increasing the amount of substances can make the reaction more vigorous and facilitate the study of the interaction mechanisms, which is reasonable for molecular dynamics. The box is large enough to contain all the molecules and free radicals. All ROS in the box are randomly distributed around the TC molecules at appropriate distances. The reason for this is to ensure that ROS do not react with TCs before the start of the simulation and to prevent TCs and ROS from being too far apart. Periodic boundary conditions are applied in all three directions of the box (x, y, z) to form an infinite periodic arrangement.

In this study, a reactive MD simulation method is adopted based on the ReaxFF field. Molecular dynamics describes the topological structure of molecules in terms of classical mechanics and relies on molecular force fields for calculations [52]. The interaction processes between atoms arise from the integration of force fields and equations of motion and do not require any assumptions about the interactions occurring between atoms, which is ideal for studying complex chemical reactions. Compared to quantum mechanics and semi-empirical methods, it has the ability to deal with large molecular systems and can save computational time significantly. In recent years, molecular force fields have developed rapidly, which has made it possible to put molecular dynamics into practical applications. Early molecular force fields such as MM2, AMBER [53], CFF and CHARMM [54] could only describe a limited number of elements and some atoms with hybrid orbitals, so their application was limited. Since the 1990s, molecular force fields such as DREIDING [55], SHARP and UFF [56] have essentially covered the entire periodic table, making molecular dynamics a powerful computational tool for scientists. However, all these fields are non-reactive force fields in which chemical bonds do not break and new bonds are not formed. To study the reaction processes between molecules and radicals the ReaxFF force field was selected for simulation. ReaxFF is one of the most accurate classical reactive force fields, having a wide range of applications in hydrocarbons [57], metal hydrides [58], metal oxides [59] and biochemical systems [60]. With the ability to accurately describe chemical reactions and bond breakage and formation, it is suitable for observing the reaction processes of ROS and TCs and exploring their interaction mechanisms. Since the emergence of ReaxFF, many scientists have optimized its parameters with different focuses for different research fields. In this paper, we aim to study the reaction processes of TCs and ROS and therefore choose the ReaxFF field studied by Karthik Ganeshan et al. [61]. This ReaxFF force field has been widely used, yielding good results [62,63,64]. Subsequently, several pre-simulations are performed and the simulation results are in good agreement with the experimental results so that the accuracy of the force field parameters can be determined. All calculations for this simulation are performed in LAMMPS.

Before the simulation, the relaxation equilibrium of the constructed reaction model is performed for 50 ps by using a Berendsen thermostat to restore the model from the non-equilibrium state to the equilibrium state. According to the molecular dynamics principle, the time-step in the MD simulations should not be too large. Otherwise, the energy changes too fast during the reaction processes, leading to the total energy of the system not being conserved and the system being unstable [40]. In the present simulation, the time step is set to 0.1 fs. After several pre-simulations, it is found that the reactions are all completed before 350 ps. Therefore, in 400 ps the computational data will have allowed all the possible reactions as well as the breaking and formation of crucial chemical bonds to occur. To collect sufficient simulation data and to ensure the reliability of simulation results, at least 20 runs are performed for each TCs and the different ROS.

## 4. Conclusions

The abuse of tetracycline antibiotics (TCs) has attracted more attention, and degradation of TCs by cold atmospheric plasmas (CAPs) is widely accepted to be an efficient and low energy consumption way. In this study, the interaction mechanisms of CAPs and TCs are deeply investigated by a reactive MD simulation based on the ReaxFF field at the atomic level. The computational data indicate that different kinds of ROS (OH radicals, O atoms, $O_{3}$ molecules and $H_{2}O_{2}$ molecules) show a series of common reactions with active functional groups (the $C_{2}$ acylamino, the $C_{4}$ dimethylamine, the $C_{6}$ methyl group, the benzene ring and the $C_{12a}$ tertiary alcohol) in TCs, such as hydroxylation, dehydroxylation, aldehyde, deamination, carbonylation, etc. Moreover, for specific functional groups in OTC, CTC and DMC, such as the $C_{5}$ secondary alcohol on OTC, the $C_{7}$ chloride group on CTC and the $C_{6}$ secondary alcohol on DMC, ROS can also undergo carbonylation and dechlorination reactions to reduce the activity of these molecules. These reactions have been observed and analyzed at the atomic level to deeply reveal the mechanisms of degradation of TCs treated by CAPs, clearly suggesting that low-temperature technology is an essential method to degrade TCs. This study summarizes the reactions of different kinds of ROS and TCs and provides the theoretical support for the experimental observation, indicating the optimization ways to improve the efficiency of degradation of TCs by CAPs in applications.

## Data Availability

The data that support the findings of this study are available from the corresponding author upon reasonable request.
